# Phase II study of capecitabine and oxaliplatin given prior to and concurrently with preoperative pelvic radiotherapy in patients with locally advanced rectal cancer

**DOI:** 10.1038/sj.bjc.6604297

**Published:** 2008-03-18

**Authors:** D Koeberle, R Burkhard, R von Moos, R Winterhalder, V Hess, F Heitzmann, T Ruhstaller, L Terraciano, J Neuweiler, G Bieri, C Rust, M Toepfer

**Affiliations:** 1 Division Oncology/Hematology, Department of Internal Medicine, Kantonsspital St Gallen, St Gallen CH-9007, Switzerland; 2 Department of Internal Medicine, Stadtspital Triemli, Zürich CH-8063, Switzerland; 3 Department of Medical Oncology, Kantonsspital Graubünden, Chur CH-7000, Switzerland; 4 Department of Internal Medicine, Kantonsspital Luzern, Luzern CH-6000, Switzerland; 5 Department of Medical Oncology, Universitätsspital Basel, Basel CH-4031, Switzerland; 6 Stadtspital Waid, Department of Internal Medicine, Zurich CH-8037, Switzerland; 7 Universitätsspital Basel, Department of Clinical Pathology, Basel CH-4031, Switzerland; 8 Kantonsspital St Gallen, Department of Pathology, St Gallen CH-9007, Switzerland; 9 Roche Pharma (Schweiz) AG, Reinach CH-4153, Switzerland; 10 Department of Radio-Oncology, Kantonsspital St Gallen, St Gallen CH-9007, Switzerland

**Keywords:** rectal cancer, radiochemotherapy, capecitabine, oxaliplatin

## Abstract

This multicentre phase II study evaluated the efficacy and safety of preoperative capecitabine plus oxaliplatin and radiotherapy (RT) in patients with locally advanced rectal cancer (T3/T4 rectal adenocarcinoma with or without nodal involvement). Treatment consisted of one cycle of XELOX (capecitabine 1000 mg m^−2^ bid on days 1–14 and oxaliplatin 130 mg m^−2^ on day 1), followed by RT (1.8 Gy fractions 5 days per week for 5 weeks) plus CAPOX (capecitabine 825 mg m^−2^ bid on days 22–35 and 43–56, and oxaliplatin 50 mg m^−2^ on days 22, 29, 43 and 50). Surgery was recommended 5 weeks after completion of chemoradiotherapy. The primary end point was pathological complete tumour response (pCR). Sixty patients were enrolled. In the intent-to-treat population, the pCR rate was 23% (95% CI: 13–36%). 58 patients underwent surgery; R0 resection was achieved in 57 (98%) patients, including all 5 patients with T4 tumours. Sphincter preservation was achieved in 49 (84%) patients. Tumour and/or nodal downstaging was observed in 39 (65%) patients. The most common grade 3/4 adverse events were diarrhoea (20%) and lymphocytopaenia (43%). Preoperative capecitabine, oxaliplatin and RT achieved encouraging rates of pCR, R0 resection, sphincter preservation and tumour downstaging in patients with locally advanced rectal cancer.

In recent years, considerable progress has been made in the treatment of locally advanced rectal cancer, mainly due to improvements in the type and quality of surgery, better staging methods and regular use of chemoradiation (CRT) or radiation therapies. Although the use of preoperative CRT for resectable rectal cancer remains a controversial issue, preoperative CRT is clearly preferred when tumour shrinkage is required before surgery, that is, in locally advanced T4 disease and low-lying tumours when sphincter preservation is attempted (Sauer *et al*, 2004; Bosset *et al*, 2006; Gérard *et al*, 2006). Furthermore, preoperative CRT improves local disease control with less toxicity compared with postoperative CRT (Sauer *et al*, 2004).

Many attempts have been made to increase the convenience and activity of preoperative 5-fluorouracil (5-FU)-based CRT. Evidence from phase II trials suggests that the oral fluoropyrimidine capecitabine (Xeloda®; F Hoffmann-La Roche Ltd, Basel, Switzerland) has similar activity to that of protracted 5-FU infusional CRT regimens (Glynne-Jones *et al*, 2006a). Combining different chemotherapy agents, such as oxaliplatin or irinotecan, with fluoropyrimidines has a clear rationale based on a plethora of data in the metastatic colorectal setting and the potential to further improve efficacy in patients receiving preoperative CRT. Oxaliplatin (Eloxatin®; Sanofi-Aventis, Bridgewater, NJ, USA) is an ideal candidate for inclusion into neoadjuvant CRT regimens because of its radiosensitising capabilities and synergy with fluoropyrimidines.

Capecitabine has been tested in combination with oxaliplatin and radiotherapy in several different regimens (for review see Glynne-Jones *et al*, 2006a). These include continuous capecitabine (7 days per week) with oxaliplatin given on days 1 and 29 (Glynne-Jones *et al*, 2006c), continuous capecitabine (5 days per week) with weekly doses of oxaliplatin (Machiels *et al*, 2005; Rutten *et al*, 2006) and discontinuous capecitabine (days 1–14 and 22–35) with oxaliplatin on days 1, 8, 22 and 29 (Roedel *et al*, 2003, 2007). The aim of the present multicentre phase II study was to evaluate the efficacy, tolerability and feasibility of preoperative capecitabine plus oxaliplatin in combination with radiotherapy as described by Roedel *et al* (2003, 2007), and to investigate the contribution of an additional single cycle of neoadjuvant capecitabine and oxaliplatin (XELOX regimen) (Díaz-Rubio *et al*, 2002; Cassidy *et al*, 2004) before the start of radiotherapy.

## MATERIALS AND METHODS

### Patient population

Patients entering the study had histologically confirmed rectal adenocarcinoma. Evidence of T3 or T4 disease with or without perirectal nodal involvement by endorectal ultrasound or magnetic resonance imaging (MRI) of the pelvis was required. Further inclusion criteria were an Eastern Cooperative Oncology Group performance status 0–2 and adequate haematological (neutrophils ⩾1.5 × 10^9^ l^−1^ and platelets ⩾100 × 10^9^ l^−1^), renal (calculated creatinine clearance >50 ml min^−1^) and liver function (serum bilirubin ⩽1.5 upper limit of normal range, liver transaminase or alkaline phosphatase concentrations ⩽2.5 upper limit of normal range). No upper age limit was defined.

Exclusion criteria were metastatic disease, previous chemotherapy for colorectal cancer or prior radiotherapy to the pelvis, history of another malignancy within the last 5 years, any contraindication to radiotherapy, clinically significant cardiac disease, malabsorption syndrome, peripheral neuropathy ⩾grade 1 according to National Cancer Institute Common Terminology Criteria for Adverse Events (NCI CTC version 3.0), serious uncontrolled infection, concomitant treatment with any nucleoside analogue, known dihydropyrimidine dehydrogenase deficiency and psychiatric disorders or conditions interfering with compliance for oral drug intake. Pregnant or lactating woman were excluded.

All patients provided written informed consent. The study protocol was approved by a local independent ethics committee and conducted in accordance with the Declaration of Helsinki.

### Pretreatment evaluation

Before study entry, all patients were assessed by a multidisciplinary team comprising medical, radiation and surgical oncologists, gastroenterologists and radiologists. Patients underwent a medical history, physical examination, biopsy, ECG and staging studies (chest X-ray, abdominal–pelvis computed tomography scan, colonoscopy and endorectal ultrasound). Pelvic MRI was optional but recommended for all patients with low-lying or T4 tumours. Complete laboratory tests included a full blood count, electrolytes, creatinine, liver transaminases, alkaline phosphatase, total bilirubin and carcinoembryonic antigen measurement.

### Treatment

#### Radiotherapy

Megavoltage equipment was used with 6–18 MV. Radiotherapy was delivered through three to four portal fields to the tumour and lymphonodal regions, and perirectal soft tissue structures at risk of microscopic disease. All patients received 45 Gy, with a daily fraction of 1.8 Gy given 5 days per week for 5 consecutive weeks. If treatment was interrupted, the dose was increased by 1–2 fractions.

#### Chemotherapy

**Figure 1:**
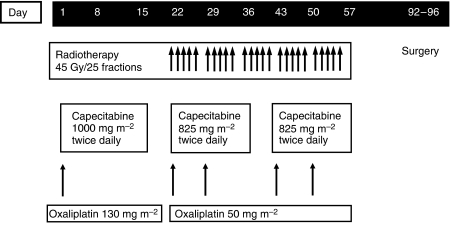
Neoadjuvant chemotherapy and concomitant chemoradiation regimen.

Treatment consisted of a single cycle of XELOX (oral capecitabine 1000 mg m^−2^ twice daily on days 1–14 plus a 2-h intravenous infusion of oxaliplatin 130 mg m^−2^ on day 1), followed by CAPOX combined with RT (capecitabine 825 mg m^−2^ twice daily on days 22–35 and 43–56, and oxaliplatin 50 mg m^−2^ on days 22, 29, 43 and 50; Figure 1). Chemotherapy and radiotherapy were interrupted if grade 3 or 4 toxicity was encountered (except for anaemia). Study treatment was restarted when toxicity had resolved to grade ⩽1. Dose reductions were required after grade 3–4 toxicity. If treatment was delayed for longer than 3 weeks, the patient was withdrawn from the study.

#### Surgery

Five to six weeks after completion of CRT, total mesorectal excision (TME) with sphincter preservation was performed whenever feasible according to standardised technique as the preferred type of radical resection. Assessment of the intended surgical procedure (TME or abdominoperineal resection) was performed by the treating surgeon before registration. Administration of adjuvant chemotherapy was left to the treating oncologist's discretion.

### Evaluation of efficacy and safety

The extent of residual tumour in the resected specimen was classified according to the TNM staging system of the American Joint Committee on Cancer/International Union Against Cancer (AJCC/UICC). Semiquantitative evaluation of histological regression was performed according to the grading criteria established by Mandard *et al* (1994) and Dworak *et al* (1997) in oesophageal carcinomas, which was subsequently adapted by Bouzourene *et al* (2002) (see Table 1 for summary).

At each site, one preselected pathologist evaluated the tumour tissue of all patients participating in the study. A second-opinion pathology review was performed in all tumours categorised as Dworak grade 2 or 3 by a pathologist from another centre participating in the study.

Safety was assessed through documenting adverse events and clinical laboratory tests performed at screening, during treatment and the surgery period. Adverse events were graded using NCI CTC version 3.0.

### Statistical analysis

The intent-to-treat population (ITT) consisted of all patients who received at least one dose of study medication whether or not they were eligible. All efficacy analyses were performed on this population. Patients not undergoing surgery or who were not evaluable for response were considered nonresponders. The safety population consisted of patients who received at least one dose of any study drug and who had a baseline assessment and at least one safety follow-up.

The primary end point was pathological complete tumour response (pCR) prospectively defined as grade 3 or 4 according to the Dworak classification (DC) system (Dworak *et al*, 1997). The pCR rate was presented with 95% confidence intervals (CIs) using the Pearson–Clopper method. Secondary end points were rate of sphincter preservation, R0 resection in patients with T4 tumours, downstaging (defined as a decrease of ⩾1 point(s) in T and/or N value) and safety.

An exploratory analysis using the Fisher exact test was performed to test for an association between the presence and absence of a complete tumour response (DC grade 3/4 *vs* 0/1/2), and site, location and size of primary tumour at screening, u/cTNM classification at screening, time between radiotherapy and surgery (⩽40 *vs* >40 days), age (⩽60 *vs* >60 years) and treatment-related lymphocytopaenia.

This study was designed as a one-stage phase II trial using pCR rate as the primary efficacy criterion. A pCR of 22% was considered acceptable and a rate of ⩽7% was ruled out as futile. With a total of 48 evaluable patients (and a response rate of at least 22%), a power of 86% and a type-I error of 4.8% was achieved. The planned sample size was increased to 60 patients to allow for dropouts.

## RESULTS

### Patient characteristics

A total of 60 patients were enrolled between March 2005 and July 2006 from six cancer centres in Switzerland. Patient characteristics are summarised in Table 2. All 60 patients were included in the safety and ITT populations, including 2 patients who were ineligible (one patient because of cT2 rectal cancer, and the other patient because of an urothelial cancer 4 years before the start of the study). Fifty-eight patients (97% of all recruited patients) received CRT and underwent surgery; one patient withdrew consent and one patient died prior to surgery.

### Dose intensity and safety

Fifty-five patients (92%) received all three cycles of capecitabine (mean relative dose intensity 97%), and 52 patients (87%) received all five planned oxaliplatin doses (mean relative dose intensity 97%). The mean relative dose intensity for capecitabine was 98% during XELOX and 96% during CAPOX-RT. For oxaliplatin, the mean relative dose intensity was 99% during XELOX and 93% during CAPOX-RT. Fifty-six patients (93%) received at least 25 fractions (45 Gy) of radiotherapy as planned.

Table 3 summarises grade 3/4 treatment-related nonhaematological toxicities presented per treatment regimen (XELOX *vs* CAPOX-RT). The most frequently occurring grade 3/4 adverse event was diarrhoea (20%); all other grade 3/4 events were uncommon (⩽5%). No grade 3/4 haematological toxicity was observed, except for lymphocytopaenia (43%). At least one serious adverse event was recorded in eight patients (13%) during the study. A total of 12 serious adverse events (20%) were reported, the most common of which were diarrhoea (*n*=5) and colitis or proctitis (*n*=2). One patient developed severe neutropaenic infection and died on day 19 after the start of neoadjuvant XELOX. Four patients (7%) had one adverse event leading to discontinuation of capecitabine, and three patients (5%) had adverse events leading to discontinuation of oxaliplatin. No patient required discontinuation of radiotherapy.

### Efficacy and surgical parameters

Surgery was performed in a total of 58 patients (TME in 47 patients (81%), abdominoperineal extirpation in 9 patients (16%) and other type of surgery in 2 patients (3%)). The median time between the end of radiotherapy and surgery was 42 days (range: 24–59 days). In 57 patients (98%), including all 5 patients with c/uT4 tumours, R0 resection was achieved and sphincter preservation was achieved in 49 patients (84%).

Comparing the baseline tumour stage with the pathological stage in the ITT population, downstaging with respect to tumour stage was observed in 28 (47%) patients, and downstaging with respect to nodal stage was observed in 29 (48%) patients. A detailed analysis is shown in Table 4.

In the ITT population, complete tumour regression (ypT0 N0, DC regression grade 4) was achieved in seven patients. An additional seven patients showed near-complete regression (DC regression grade 3) with only very few detectable tumour cells as assessed by two independent pathologists. According to predefined criteria, the pCR rate was therefore 23% (95% CI: 13–36%). The corresponding pCR rate according to the Mandard regression grading system (grades 1 and 2) was 27% (grade 1, seven patients; grade 2, nine patients).

A second-opinion review of all specimens rated as DC grade 2 or 3 was necessary in 33 cases (57%). After the second opinion, the final DC grading remained the same in 27 cases (82%), downgrading was deemed necessary in 5 cases (15%) and upgrading in 1 case (3%). Both tumour regression scales were compared using the final DC grades and Mandard grades. The scales seemed to correspond well; all patients with DC grade 0 reported Mandard-tumour regression (M-TR) grade 5, 90% of patients with DC grade 1 reported M-TR grade 4, 74% of patients with DC grade 2 reported M-TR grade 3, 86% of patients with DC grade 3 reported M-TR grade 2 and all patients with DC grade 4 reported M-TR grade 1.

According to an exploratory subgroup analysis, only upper location of the primary tumour (between 10 and 12 cm from anal verge) was found to be negatively correlated with pCR (*P*=0.0504).

## DISCUSSION

Pathological complete tumour response rates between 10 and 30% have been observed with combined preoperative chemotherapy and radiotherapy protocols. Pathological complete tumour response is a reliable and reproducible surrogate for tumour response and is linked to improved outcome (Roh *et al*, 2004; Roedel *et al*, 2005). Although achievement of a pCR is not the primary goal of neoadjuvant therapy, it has become a commonly used end point in many phase II trials aiming to improve the efficacy of rectal cancer treatment.

In the present trial, we are able to demonstrate a pCR in 23% of patients, defined as grades 3 and 4 according to the Dworak classification (Dworak *et al*, 1997) following preoperative therapy with a single cycle of XELOX and two further cycles of CAPOX given with radiotherapy. Recently, several different tumour regression scales (Mandard *et al*, 1994; Dworak *et al*, 1997; Bouzourene *et al*, 2002; Wheeler *et al*, 2004) have been proposed for the measurement of regression after preoperative therapies independent of the ypTNM stage. Besides several differences in categorisation of tumour regression, all of the scales acknowledge a distinctive group of tumours with only microscopic foci of remaining tumour cells. We have grouped patients with sterilised primary tumours and lymph nodes (DC grade 4) together with DC grade 3 tumours. This is based on the observation that single residual tumour cells confer a significantly lower local relapse rate and a better prognosis (Wheeler *et al*, 2004) than tumours with remaining dominant disease.

The second-opinion review was used for all specimens rated to be either DC grade 2 or 3 by the first pathologist. A high concordance rate between independent pathologists of 82% suggests a reasonable capability to discriminate between very few and difficult-to-find tumour cells (DC grade 3) from easy-to-find few tumour cells or groups of tumour cells (DC grade 2). In addition, applying the regression grading classification of Mandard *et al* (1994) adapted for rectal cancer (Bouzourene *et al*, 2002) and combining Mandard's grade 1 (absence of residual tumour cells) and grade 2 (rare residual tumour cells), complete tumour response was achieved in 27% of patients. These almost identical results from two different scoring systems indicate a high reproducibility in defining near-complete and complete sterilisation of tumour cells.

Furthermore, and an important point with regard to prognosis, we observed nodal downstaging in 48% of patients. The number of tumour-infiltrated lymph nodes (ypN status) following preoperative radiochemotherapy is a strong and independent prognostic factor for survival. Sterilising lymph nodes reflects the impact of effective neoadjuvant treatment and consistently translates into improved long-term outcome (Bouzourene *et al*, 2002; Chan *et al*, 2005; Chapet *et al*, 2005; Roedel *et al*, 2005).

In 98% of the 58 patients who underwent surgery and in all patients with T4 rectal cancer, R0 resection was possible. Sphincter preservation was achieved in 84% of patients. This appears remarkable as 35% of our patients had low-lying tumours (0–5 cm from anal verge).

The most common nonhaematological toxicity in our trial was grade 3 or 4 diarrhoea, which occurred overall in 20% of patients (10% during XELOX and 10% during CAPOX-RT). This rate is slightly higher than that reported by Roedel *et al* (2003, 2007) and suggests that the additional cycle of XELOX increased the toxicity of preoperative CRT. All other toxicities were in the range of other trials with the exception of grade 3 or 4 lymphocytopaenia. Lymphocytopaenia is a negative prognostic factor in cancer patients and can be induced by either chemotherapy or pelvic radiotherapy. Decline in total lymphocyte counts is obviously an underreported toxicity and seems to be negatively correlated with tumour regression following pelvic radiotherapy (Lissoni *et al*, 2005).

The addition of a single chemotherapy cycle before CRT does not appear to have substantially enhanced the overall antitumour activity and should, therefore, not be considered as an important treatment element. In our trial, we added this chemotherapy cycle primarily to assure early start of therapy. Even though many patients reported improvement in symptoms before starting CRT (data not shown), we did not consider achieving a relevant downsizing effect with a single chemotherapy cycle.

The use of neoadjuvant chemotherapy prior to preoperative CRT in rectal cancer patients is a matter of debate (Glynne-Jones and Sebag-Montefiore, 2006; Glynne-Jones *et al*, 2006b) primarily because satisfactory local control rates can be achieved with preoperative CRT alone. Chau *et al* (2006) questioned this position by adding four cycles of neoadjuvant capecitabine and oxaliplatin before CRT with capecitabine in their trial. Most patients (86%) had symptomatic responses, and the radiological response rate measured by MRI was 88%. Pathological complete tumour response was achieved in 24% of patients, which is clearly superior to the 11% DC regression grade 4 in our trial. However, 4 out of 77 patients died during neoadjuvant chemotherapy. In the absence of a randomised phase III trial proving superior outcome, the addition of primary chemotherapy to CRT should be only used in the context of clinical trials.

A different treatment strategy, in an attempt to increase the quantity of systemic treatment, was studied by Roedel *et al* (2007). After preoperative capecitabine/oxaliplatin radiotherapy, 60% of patients received all four cycles of adjuvant capecitabine/oxaliplatin underlining the feasibility of delivering adequate doses of postoperative combination chemotherapy in rectal cancer patients.

In conclusion, we demonstrated that preoperative XELOX followed by CAPOX-RT is feasible with manageable toxicity and results in encouragingly high rates of pCR, R0 resection, sphincter preservation and tumour downstaging in patients with locally advanced rectal cancer. More importantly, we were able to replicate, and thus confirm the findings from Roedel *et al* (2003, 2007) in a multicentre setting in Switzerland.

## Figures and Tables

**Table 1:** Histological tumour regression grading systems

**Grade**	**Definition**
Dworak *et al* (1997)	
0	No regression
1	Dominant tumour mass with obvious fibrosis and/or vasculopathy
2	Dominantly fibrotic changes with few tumour cells or groups (easy to find)
3	Very few (difficult-to-find microscopically) tumour cells in fibrotic tissue with or without mucous substance
4	No tumour cells, only fibrotic mass (total regression or response)
	
Mandard *et al* (1994) (adapted by Bouzourene *et al*, 2002)	
5	Absence of regressive changes
4	Residual cancer outgrowing fibrosis
3	Increase in the number of residual cancer cells, but fibrosis still predominant
2	Presence of rare residual cancer cells scattered through the fibrosis
1	Complete regression, absence of histologically identifiable residual cancer and fibrosis extending through the different layers of the rectal wall, with or without granuloma

**Table 2:** Patient characteristics (*N*=60)

**Characteristic**	**No. of patients**	**%**
*Sex*		
Male	46	77
Female	14	23
		
*Age, years*		
Median (range)	61 (35–76)	
		
*u/cTNM classification*		
T stage 2	1	2
T stage 3	53	88
T stage 4	6	10
		
N stage 0	13	22
N stage 1	44	73
N stage 2	3	5
		
*Location of tumour^a^*		
Lower rectum (0–5 cm)	22	37
Middle rectum (5.1–10 cm)	30	50
Upper rectum (10.1–12 cm)	17	28
		
*Size of primary tumour (mm)*		
Median (range)	50 (16–140)	
		
Infiltration of sphincter muscle	6	10
		
*Intended type of surgery*		
Total mesorectal excision	46	77
Abdominoperineal resection	14	23

a More than one location per patient possible.

**Table 3:** Most frequently reported nonhaematological treatment-related adverse events (*N*=60)

	**Toxicity according to NCI CTC grade (% of patients)**					
	**Cycle 1 (XELOX pre-radiotherapy)**			**Cycles 2 and 3 (CAPOX radiotherapy)**		
**Adverse event**	**1/2**	**3**	**4**	**1/2**	**3**	**4**
Diarrhoea	12	10	—	38	10	—
Nausea/vomiting	29	3	—	15	—	—
Mucositis	—	2	—	27	3	—
Bleeding	2	—	—	3	2	—
Constipation	7	—	—	3	—	—
Pain	7	3	—	40	2	—
Fatigue	15	2	—	28	—	—
Infection	—	—	2	12	—	—
Hypokalaemia	—	2	—	—	2	—
Anorexia	6	2	—	8	2	—
Neuropathy	27	—	—	30	—	—
Dysuria	5	—	—	32	—	—
Syncope	—	2	—	—	—	—
Dyspnoea	3	—	—	3	2	—
Hand–foot syndrome	2	—	—	6	2	—
Dermatitis	3	—	—	3	—	—
Rash	3	—	—	3	—	—
Thrombosis	—	—	—	—	3	—

NCI CTC=National Cancer Institute Common Terminology Criteria.

**Table 4:** Preoperative T/N stage compared with pathological T/N stage (*N*=58)

**Baseline staging**	**pT0**	**pTis**	**pT1**	**pT2**	**pT3**	**pT4**	**pN0**	**pN1**	**pN2**	**pNx**
uT2	—	—	1	—	—	—	—	—	—	—
										
uT3	5	1	1	15	28	2	—	—	—	—
										
uT4	1	—	—	3	1	—	—	—	—	—
										
uN0	—	—	—	—	—	—	7	3	2	1
										
uN1	—	—	—	—	—	—	28	6	7	1
										
uN2	—	—	—	—	—	—	1	—	2	—
